# Computer‐based simulation to reduce EHR‐related chemotherapy ordering errors

**DOI:** 10.1002/cam4.3496

**Published:** 2020-10-01

**Authors:** Kirk D. Wyatt, Elizabeth B. Freedman, Grace M. Arteaga, Vilmarie Rodriguez, Deepti M. Warad

**Affiliations:** ^1^ Division of Pediatric Hematology/Oncology Mayo Clinic Rochester MN USA; ^2^ Division of Pharmacy Mayo Clinic Rochester MN USA; ^3^ Division of Pediatric Critical Care Mayo Clinic Rochester MN USA

**Keywords:** electronic health records, high fidelity simulation training, medical informatics, patient harm, patient safety, simulation training

## Abstract

**Background:**

The electronic health record (EHR) is a contributor to serious patient harm occurring within a sociotechnical system. Chemotherapy ordering is a high‐risk task due to the complex nature of ordering workflows and potential detrimental effects if wrong chemotherapeutic doses are administered. Many chemotherapy ordering errors cannot be mitigated through systems‐based changes due to the limited extent to which individual institutions are able to customize proprietary EHR software. We hypothesized that simulation‐based training could improve providers’ ability to identify and mitigate common chemotherapy ordering errors.

**Methods:**

Pediatric hematology/oncology providers voluntarily participated in simulations using an EHR testing (“Playground”) environment. The number of safety risks identified and mitigated by each provider at baseline was recorded. Risks were reviewed one‐on‐one after initial simulations and at a group “lunch‐and‐learn” session. At three‐month follow‐up, repeat simulations assessed for improvements in error identification and mitigation, and providers were surveyed about prevention of real‐life safety events.

**Results:**

The 8 participating providers identified and mitigated an average of 5.5 out of 10 safety risks during the initial simulation, compared 7.4 safety risks at the follow up simulation (p=0.030). Two of the providers (25%) reported preventing at least one real‐world patient safety event in the clinical setting as a result of the initial training session.

**Conclusions:**

Simulation‐based training may reduce providers’ susceptibility to chemotherapy ordering safety vulnerabilities within the EHR. This approach may be used when systems‐based EHR improvements are not feasible due to limited ability to customize local instances of proprietary EHR software.

## Graphical abstract

Simulation‐based training appears effective for reducing providers’ susceptibility to common safety risks within the electronic health record. Simulation may facilitate error reduction in situations where systems‐based changes are not feasible.

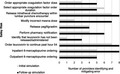

## 1 BACKGROUND

Although EHRs have been designed to overcome safety risks associated with paper‐based ordering, modern electronic health records (EHRs) have introduced new types of errors, which have been associated with serious patient harm.^1^, ^2^ Chemotherapy ordering is a particularly high‐risk task due to the complexity of sequenced multidrug regimens and the toxic effects of chemotherapeutic agents, coupled with their narrow therapeutic index.^3^

In May 2018, our institution, Mayo Clinic—Rochester, underwent an EHR transition. As a consequence, the Pediatric Hematology/Oncology practice had to adapt from homegrown chemotherapy ordering software to a module within a third‐party vended EHR (Beacon, Epic Systems, Verona, WI). Challenges during this transition included adapting to new and unfamiliar workflows, learning to navigate a new user interface, and developing workarounds to overcome software shortcomings.

Following the transition, we recognized new patterns of recurrent chemotherapy ordering errors that did not previously occur with the old software. In many cases, these errors arose because new workflows demanded by the EHR software conflicted with provider expectations, were unintuitive or were difficult for providers to adopt. Although we encouraged patient safety event reporting, it is well‐recognized that event reporting is subject to significant reporting bias. As a consequence, we were unable to accurately quantify the frequency with which these errors occurred. Some of these errors were easily mitigated through a systems‐based approach which involved reconfiguration of the chemotherapy ordering module. This approach was taken on account of decades of human factors research suggesting that error reduction is best achieved by improving the system within which humans operate rather than trying to change human behavior.^4^ Other errors could not be prevented using this approach because specific aspects of the proprietary software could not be reconfigured at the institution level, or reconfiguration would lead to adverse downstream consequences for other aspects of care. These latter errors were of particular interest to us as they exemplify the complex sociotechnical system that healthcare is delivered within.

The use of simulation education in healthcare has been widely adopted to improve patient safety. Although simulation can be used by learners of all levels, simulation‐based training has become a key component of graduate medical education programs.^5^ Simulation allows providers to practice high‐risk scenarios in a non‐intimidating and psychologically safe environment with no risk of actual patient harm. Components of effective simulation include deliberate practice, effective debriefing and individualized learning.^6^ For many skills, simulation‐based training approaches appear to be more effective than traditional education methods.^7^ In light of the evidence supporting simulation and the advantages of this approach, use of EHR‐based simulation to improve patient safety has been advocated.^8^ EHR‐based simulation permits deliberate practice without risk of patient harm, can be followed by debriefing, provides psychological safety—enhancing the learning experience—and can be individualized.

We aimed to reduce the proportion of safety hazards within the EHR that providers were susceptible to through the implementation of an EHR training program designed to simulate common threats to patient safety, including ordering of clotting factor as well as chemotherapy by the oral, intravenous, and intrathecal routes.

## 2 METHODS

### 2.1 Patient safety approach

We have adopted the systems‐based approach to patient safety at our institution.^4^ At the core of this approach is the affirmation that all health care providers strive to provide safe patient care. We acknowledge that numerous aspects of the sociotechnical system that providers operate in (e.g., busy clinical load, environmental noise, pager, and phone calls, work interruptions, the EHR) all contribute to medical errors. The most effective approach to improve patient safety is to determine the system‐related errors that contribute to safety events and improve the system to reduce the risk of error, rather than trying to directly modify human behavior. This necessitates a culture of open inquiry and a focus on human (behavioral) factors.^4^ Although we preferred to modify our EHR configuration to reduce the risk of errors, we were limited in our ability to modify the functionality of the third‐party vended EHR in use at our institution. To overcome these limitations, we attempted to address providers’ use of the EHR in a manner inconsistent with the system designers’ expectations using simulated vignettes.

### 2.2 Identification of safety risks

A number of methods were used to identify aspects of the EHR, which posed a safety risk. All staff, including staff physicians, trainee physicians, advanced practice providers, nurses, and desk operations staff, were encouraged to report incidents of system malfunction and seek technical support from the Information Technology Help Desk (HD). Staff were also encouraged to report all unsafe situations and patient safety events to a voluntary, confidential, nonpunitive event reporting system (ERS). Additionally, scheduled and *ad hoc* discussions took place in‐person, by phone, and electronically between clinicians, division leadership and information technology to review newly emergent safety concerns. After a review of HD and ERS reports and discussions with information technology liaisons, a list of ten key safety vulnerabilities affecting our pediatric hematology‐oncology practice was compiled (see Table 1 and Appendix S1). We selectively chose to address patient safety events that were not easily amenable to rapidly deployable systems‐based interventions. The selected vulnerabilities (Table 1) were prioritized because they were either identified to contribute to multiply‐recurrent patient safety events or were expected to affect a large proportion of providers. Five realistic patient medication ordering scenarios, which included the 10 safety vulnerabilities, were devised. Four of the scenarios pertained to chemotherapy ordering, and one related to coagulation factor infusion ordering. The scenarios and related safety risks are summarized in Table 1 and described in detail in Appendix S1.

**TABLE 1:** Summary of scenarios^a^

Scenario	Safety risk
Factor ordering	Ordering appropriate infusion dose
	Ordering appropriate infusion duration
Intrathecal chemotherapy ordering	“Release” intrathecal chemotherapy within correct encounter
IV chemotherapy ordering	Identify incorrect mesna dose
	“Release” outpatient pegfilgrastim
	Notify pharmacy of signed chemotherapy orders
High dose methotrexate	Identify leucovorin has not been “released”
	Identify leucovorin must be reordered to continue
6‐mercaptopurine ordering	Place two separate inpatient orders using “user specified” frequency
	Write oral 6‐mercaptopurine outpatient prescription without conflicting sig

^a^ See Appendix S1 for details of the individual scenarios.

### 2.3 Scenario programming

Our information technology liaisons—who received proprietary training from the EHR manufacturer—programmed the scenarios in the “Playground” environment, which is a testing and training virtual environment of the EHR (Epic Systems). The “Playground” environment is available to all EHR users at our institution at any time for additional practice and training. This environment had the same look, feel and functionality as the “Production” environment used for patient care, except that the patients were fictitious and orders placed were nonconsequential. One mock patient record, within which all scenarios could be deployed, was generated by applying chemotherapy “Treatment Plans” to the patient and performing the necessary steps to recreate the scenario as would be done in a real clinical encounter. Details of the scenarios are included in Appendix S1. To generate the scenarios, additional steps were required within the “Playground” simulation environment. For the intravenous chemotherapy ordering scenario, an erroneous mesna dose was entered and all orders were single‐signed. For the high dose methotrexate scenario, all chemotherapy orders were signed and the methotrexate was released but the leucovorin order was not released. For the oral 6‐mercaptopurine ordering scenario, a “treatment plan” including inpatient and outpatient 6‐mercaptopurine was simply applied to the patient record without any modifications. The patient's body surface area was chosen to ensure that the appropriate oral 6‐mercaptopurine dose would require two different dosages to be given in each week. The patient record was duplicated so that multiple providers could perform simulations simultaneously on different—but identical—patient records. Every night, the “test patient” records would revert to their original state so that simulations could be repeated on subsequent days without the need to be manually reset to the original state. This functionality is standard and should be available at other institutions using the same EHR.

### 2.4 Human subjects’ protection

As a quality improvement project, this study was exempt from Institutional Review Board oversight. All participants were informed that their participation was voluntary. Participants were informed that data would be shared externally only in aggregate form.

### 2.5 Participant selection

All providers within the Division of Pediatric Hematology/Oncology at Mayo Clinic, Rochester, Minnesota, including seven attending physicians, two fellows and one nurse practitioner, were given an overview of the simulation‐based training and sent a follow‐up email explaining the simulation process. Providers were invited to participate and informed that participation was completely voluntary. Division leadership endorsed the simulation‐based training and authorized blocking of clinical calendars to facilitate the simulations so that provider availability was not a barrier to participation.

### 2.6 Data collection

The main outcome of interest was the proportion of patient safety risks (out of 10) that providers identified and mitigated before and after the intervention. Secondary outcomes included provider self‐reporting of whether they had applied anything they had learned in the previous simulation, avoided an error as a result of the simulation training and whether they would be interested in participating in similar training in the future (with response values of yes, no or unsure for all three questions).

### 2.7 Initial simulations

A simulation facilitator (KDW) arranged a 1‐hour appointment to meet with each participant privately in their office, or in a conference room if their office was shared or in a location distant from the clinic. Offices included a desk with a computer which was attached to either one or two widescreen monitors. Offices were located within the clinic but were down the hall from the patient rooms. The conference room included a large‐screen TV connected to a computer and a large rectangular conference room table. We considered conducting simulations within a shared workroom, which includes 11 computers (seven along opposite walls and four on a center “island”), is located adjacent to patient rooms, and is where most providers perform chemotherapy ordering tasks. However, this was not done for two reasons. For one, we sought to provide a psychologically safe space for providers to perform the simulations, and co‐workers would overhear interactions with the facilitator if the simulations were conducted in the workroom. Additionally, conducting the simulations in front of other providers would unblind future participants to the content of the scenarios and the safety risks. For these reasons, simulations were conducted in private.

Participants were blinded to the scenarios until the simulation began. For each scenario, the provider was read a scripted patient vignette and asked to complete specific tasks within the EHR simulation environment. To make the simulation as realistic as possible and to not draw specific attention to the safety risks of interest, the scenarios often required performance of extraneous tasks that were not associated with safety risks but were realistic within the scenario. For example, a provider was asked to sign an entire “day” of chemotherapy including multiple orders, even though only one or two of the orders was of interest as a safety risk. When necessary, providers were given printed copies of supplemental documents often used in the clinic setting during chemotherapy ordering (e.g., treatment protocol reference documents). Furthermore, providers were encouraged to use any other resources they would normally use when ordering chemotherapy (e.g., handheld calculator).

The simulation facilitator was not allowed to provide guidance to participants and silently observed while participants completed tasks. If participants directly asked the facilitator for assistance, the facilitator reminded participants that he could not provide any guidance and instructed participants to make their best attempt at completing the tasks. The facilitator marked on a checklist how many safety risks within each scenario were or were not identified and mitigated by the participant to quantitatively measure the primary endpoint of interest. After each scenario, the facilitator performed a debriefing where he shared the safety risks with the participant and walked through the scenario, demonstrating the steps required to mitigate the safety risk.

Following the simulation, each provider was sent a confidential email summarizing each safety risk and providing feedback on which safety risks the provider identified and mitigated during the initial simulation.

### 2.8 Group session

After each participant completed an initial individual simulation session, an all‐hands “lunch‐and‐learn” group session to which all providers were invited was conducted to review the safety risks and reinforce the steps needed to mitigate them. An information technology liaison participated in the meeting to clarify participant questions regarding navigation of the EHR and workflows.

### 2.9 Follow‐up simulations

To assess whether providers were better able to identify and mitigate safety risks following initial simulations and the “lunch‐and‐learn” session, a follow‐up observation was arranged. We scheduled follow‐up sessions approximately 3 months after the initial simulation to assess retention in long‐term memory at a time remote from the initial simulation, as has been described for simulation education previously.^9^, ^10^ The facilitator met with participants one‐on‐one, and the follow‐up simulations were conducted identically to the initial sessions except that providers were also verbally surveyed with three questions: (a) Have you applied anything you learned in the previous simulation with real patients? (b) Have you prevented any patient safety events as a result of these training sessions? (c) Are you interested in this type of simulated learning again in the future?

Due to the coronavirus (COVID‐19) pandemic in spring 2020, two of the follow‐up observations were conducted remotely—rather than in person—using teleconferencing software with screen‐sharing capabilities.

### 2.10 Statistical analysis

Data were entered into JMP (Sas Institute) for analysis. The difference in number of events identified by providers following the initial simulation and lunch‐and‐learn session was assessed using a paired t‐test with two‐tailed *p*‐values < 0.05 considered significant.

## 3 RESULTS

**FIGURE 1:**
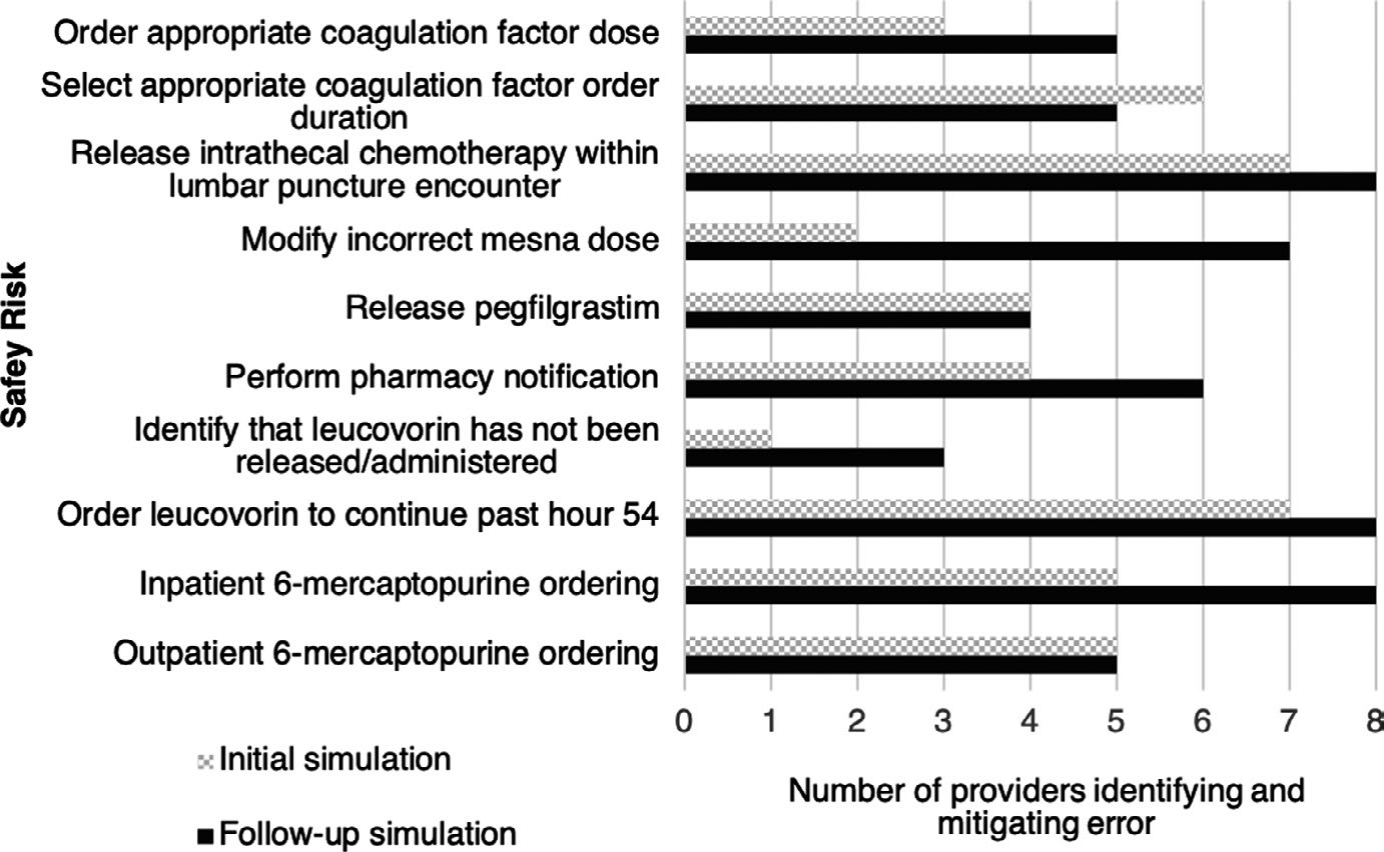
Provider identification and mitigation of errors during initial and follow‐up simulation assessments (n = 8)

Eight providers, the majority of whom were attending physicians, voluntarily participated in initial and follow‐up simulations. Follow‐up simulations occurred at a median of 95.5 days (range 84‐155) following initial simulations. The average provider identified and mitigated 5.5 out of 10 safety risks (standard deviation 2.1) during the initial simulation. At the follow‐up simulation, the average provider identified and mitigated 7.4 out of 10 safety risks (standard deviation 1.1), for an average absolute difference of 1.8 more (18%) safety risks identified following initial simulation (95% confidence interval 0.24‐3.51; *p* = 0.03; Figure 1). Qualitatively, we observed that some participants initially committed errors during follow‐up scenarios and then immediately recognized the safety vulnerability and recovered the error.

Three of the providers (38%) indicated that they had applied at least one thing they learned in the previous simulation with a real patient, three providers were unsure and two indicated that they did not. Two of the providers (25%) reported at the follow‐up assessment that they had prevented at least one real‐life patient error as a result of the training session, three providers were unsure, and three indicated that they had not. All but one (88%) were interested in doing additional simulated EHR safety learning in the future.

## 4 DISCUSSION

We describe a process for identifying, assessing, simulating, and preventing common safety risks associated with chemotherapy ordering in clinical practice using simulation. We observed that providers’ ability to identify and mitigate patient safety risks was improved following simulation‐based training. Initial assessments revealed that providers were, on average, susceptible to nearly half of the identified safety risks approximately a year and a half following the initial EHR go‐live. The persistence of safety risks following the initial go‐live period emphasizes the need for ongoing surveillance and vigilance for EHR‐related safety risks. Indeed, we observed that providers remained vulnerable to over one quarter of safety risks at the time of the follow‐up simulations. Safety risks may relate to local‐level configuration of the EHR, or they may be intrinsic to the EHR vendor's software design.

We have demonstrated feasibility of performing simulations in‐person and remotely when necessary. A significant advantage of the simulation approach is that it allows for an objective assessment of providers’ susceptibility to real‐world errors in a psychologically safe and realistic environment associated with high‐fidelity and real‐time feedback. In particular, it was significant that the EHR environment within which the simulations took place were exactly identical to the environment within which providers conducted patient care (with the exception that the software was labeled as the “Playground” environment). The simulations were low in cost to conduct and used resources that were already at our disposal.

Many centers utilize *in situ* simulations within the clinical care environment to facilitate timely and realistic simulation‐based learning.^11^ Although our simulations were conducted during time set aside for the simulations, they occurred in the physical setting of the clinic. Furthermore, simulations were frequently accompanied by the common interruptions attendant to clinical practice, such as pager alerts and phone calls. Core components of simulation‐based programs have been proposed. Simulations should be of high fidelity and incorporate deliberate practice, feedback, outcome measurement, and transfer to practice.^6^, ^12^ In addition to providing an opportunity to practice skills, *in situ* simulations may also provide a means by which previously unidentified latent safety threats can be identified.^13^

EHR‐based simulations have been used in various settings to identify safety risks. For example, the Leapfrog evaluation tool allows institutions to evaluate their EHR instance to identify unmitigated safety risks related to order entry and clinical decision support. A study across 41 pediatric hospitals identified that EHR instances varied widely in identification of potential medication errors. On average, over one third of errors were not identified.^14^

Individual user‐level simulations also appear effective at measuring providers’ susceptibility to error and reducing the incidence of errors. An intensive care unit EHR simulation at Oregon Health and Science University, using the same EHR used at our institution, assessed whether providers identified 14 safety risks. Although there was a wide range of error recognition across participants, the average participant failed to identify 59% of patient safety risks.^15^ In a follow‐up study including users who repeated simulations over a month later, users identified a greater proportion of safety issues on repeat simulation, suggesting that participation in the initial simulation improved performance at follow up.^16^

Another simulation‐based study at Children's Hospital of Philadelphia—also using the same EHR—observed that pediatric residents were more likely to utilize a data visualization tool following simulation‐based training.^17^ This suggested that simulation‐based methods can effect changes in providers’ EHR usage behaviors.

In the course of our evaluation of EHR‐related safety risks, we favored reducing risk by reconfiguring the current EHR system rather than by attempting to change human behavior. In some cases, systems‐based changes to the EHR were feasible and within reach. In contrast, other safety risks remained that could not be remedied through system reconfiguration. These risks for which we were unable to implement systems‐based changes were the most unsatisfying.

Improving EHR design is an important patient safety priority. In large part, the safety vulnerabilities we identified reflect the challenge of developing an integrated, universally deployable and scalable software suite that is capable of facilitating patient care within an incredibly complex and unpredictable sociotechnical system. Computer software performs well when it manages clearly defined processes, which follow a predictable routine. However, in systems where exceptions seemingly outstrip instances where a general rule is followed, the system's performance degrades. In our experience, many of the safety risks we observed represented “fringe” cases where the system had to be modified to accommodate a specific situation that, while routine within our scope of clinical practice, is outside of the norm for how most medications are prepared or administered. Chemotherapy also presents many unique challenges, because medications may be administered within the hospital, in an outpatient infusion center and at home over multiple days and in a particular sequence. Indeed, we have observed the notion of “encounter”‐based care that EHRs rely on for billing and other administrative purposes to be at odds with the reality that health care occurs across a longitudinal continuum of care.

Our need to rely on “soft” solutions (e.g., training), rather than “hard” solutions (e.g., system reconfiguration) highlights a challenge that is faced by many institutions who rely on third‐party commercially vended EHRs. In an ideal world, individual institutions would be able to customize and reconfigure every aspect of the EHR with ease to streamline institutional workflows and facilitate patient safety while maintaining the capability of sharing patient information between institutions when desired. However, many aspects of the EHR cannot be customized by end‐users, or they can only be customized to a certain extent that is dictated by the EHR vendor. This represents a major challenge, especially when the existing design is judged by users to be confusing or unintuitive. We anticipate that the widespread adoption of health information exchange application programming interfaces (APIs) and interoperability standards, including HL7 SMART on FHIR,^18^ will improve patient safety by allowing end‐users to customize their systems and interfaces in ways that improve patient safety. The ability to “plug‐and‐play” with SMART on FHIR modules may also permit the rapid dissemination of best practices for system interface and workflow design by providing an open exchange for evidence‐based features which improve patient safety in a manner that is EHR‐agnostic.

Data standards have the potential to improve chemotherapy ordering workflows. Institutions implementing a new EHR often must build each chemotherapy treatment plan manually. This requires a significant amount of resources for programming and validation, and this work is duplicated across multiple institutions that use the same chemotherapy regimens. Institutions implementing a new EHR may not fully understand the best practices and limitations relating to a new EHR until after the “go‐live” date, at which point changes require significant resources to reconfigure. Indeed, at our institution, we identified a number of treatment plan design decisions which, in retrospect, were suboptimal. This required significant time and effort in the months and years following our go‐live to reconfigure them. We advocate for data standards organizations, including HL7, to collaborate with EHR vendors to implement an interoperable approach to chemotherapy orders. We further propose partnership with pediatric oncology cooperative groups to facilitate the dissemination of chemotherapy protocols so that they be deployed by any institution, using any EHR, with industry best practices for safe and efficient care in mind. With improved inter‐institution collaboration, institutions can learn from each other's experiences and improve practices for local level EHR configuration and implementation.

Quality improvement projects are more likely to be successful if they focus on measurement of effect size.^19^ Yet, one challenge to patient safety quality improvement projects is the ability to measure the impact of the intervention. Voluntary patient safety event reporting is an industry best‐practice, but less than one in ten events are reported through these mechanisms.^20^, ^21^ Variable reporting by frontline staff can introduce reporting bias into pre‐post analyses, and small numbers of reports—which occurs with latent errors—may limit statistical power to determine whether an intervention has reduced the incidence of specific types of incidents. Furthermore, lack of a standard taxonomy for categorizing similar EHR‐related patient safety events limits our ability to easily identify similar and recurrent events.

One strength of this study is the inclusion of multiple sessions including feedback to reinforce key concepts (i.e., initial simulation, follow‐up email report, “lunch and learn” session, follow‐up simulation), with two of these instances being one‐on‐one, face‐to‐face sessions. Similarly, in addition to chemotherapy ordering in routine clinical practice, there were three opportunities to practice using the training environment (initial simulation, “lunch and learn” session, follow‐up simulation). We also included a variety of safety risks with varying level of difficulty in identification (see Table 1 and Appendix S1) and demonstrated the feasibility of conducting simulations remotely due to the COVID‐19 pandemic. One advantage of the simulation‐based approach is that each provider is presented with multiple unsafe situations and is systematically assessed for their handling of the unsafe situation. Performing pre‐post assessments on the same participants eliminates certain types of bias that may be introduced using nonmatched samples. However, we cannot rule out the possibility that improvements in providers’ ability to identify and mitigate safety risks are attributable to increased familiarity with the software over the 3 months that spanned between assessments. We felt that this was unlikely, however, because the initial assessments identified that providers remained susceptible to a number of safety vulnerabilities a year and a half following the EHR transition. Additional limitations of this study include a small sample size, implementation at one specific institution, the use of only one follow‐up assessment, and lack of a control group. Inclusion of a control group would help to isolate the effect of time and ongoing experience with the EHR. It is unclear whether this approach would be feasible or effective in larger practices.

Because 88% of providers were interested in similar simulations in the future, and the simulations were effective for reducing providers’ susceptibility for errors, we plan to conduct subsequent rounds of simulation. These subsequent rounds can include safety risks that a large proportion of providers remained susceptible to after the initial simulation‐based training, incorporate newly identified safety risks, and include other staff members who are involved in the chemotherapy ordering process (e.g., nurses, pharmacists).

## 5 CONCLUSION

Chemotherapy ordering scenarios with potential for error exist in all EHR environments. Simulation‐based approaches may reduce error rates and facilitate error recovery, but they are not a substitute for systems‐based solutions. Data standards‐based approaches may further the ability of individual institutions to customize their EHR instances to improve patient safety.

## 6 AUTHOR CONTRIBUTIONS

Kirk D. Wyatt: conceptualization, data curation, formal analysis, investigation, visualization, writing – original draft. Elizabeth B. Freedman: resources, software, writing – review and editing. Grace M. Arteaga: supervision, writing – review and editing. Vilmarie Rodriguez: conceptualization, methodology, writing – review and editing. Deepti M. Warad: conceptualization, methodology, writing – review and editing

## CONFLICTS OF INTEREST

The authors have no conflicts of interest to declare.

## Supporting information

Appendix S1

## Data Availability

To maintain participant confidentiality, the raw data set is not available. Aggregate data used for the primary analysis can be found in Figure 1.
