# Supplementary material for: Computer‐based simulation to reduce EHR‐related chemotherapy ordering errors
Source: Cancer Med. 2020 Oct 1;9(23):8844–51. doi: 10.1002/cam4.3496 (PMC7724293; doi:10.1002/cam4.3496)
Supplement: Supplementary file 1 — Appendix S1 [file CAM4-9-8844-s001.docx]

**Supplemental Appendix 1.** Medication ordering scenarios

1. **Scenario 1: Factor Ordering**
   1. **Vignette:** The patient has Hemophilia B. He is currently undergoing surgery and has already received a factor infusion which has raised his Factor IX level to 100%. He now requires continuous infusion of Factor IX at a dose of 6 units/kg/hr, which should continue for 48 hours postoperatively.
   2. **Task:** Order continuous Factor IX infusion at a rate of 6 units/kg/hr.
   3. **Safety risk explanation**
      1. **Ordering the appropriate dose:** Providers seeking to order continuous factor replacement infusion are unable to order in units of “units/kg/hr,” as they would expect to because of practice decisions related to dispensing. Instead, providers must order a bag containing enough factor replacement for eight hours and specify that the total dose must be given over eight hours. Reminder text is included at the top of each order to explain this unintuitive workflow to providers; however, for some orders, the text is truncated and providers must click a hyperlink to expand and view the entire explanation. In this scenario, the provider must order a total dose of 48 units/kg, which will be administered over eight hours (6 units/kg/hr x 8 hours).
      2. **Modify order duration to 48 hours or greater:** The order screen included two fields indicating a duration of time.
         One read as follows:
         *For: ___ hours/days*
         Another read as follows and was pre-filled:
         *Administer over: 8 hours*
         The latter field specifies that the total ordered dose (48 units/kg) should be administered over eight hours (i.e., at a rate of 6 units/kg/hr) and the former field specifies how long the order should be active (in this case, 48 hours). Because it may not be clear to providers what each duration refers to, some providers may inappropriately change the “administer over” time to 48 hours, resulting in an inadequate infusion rate.
   4. **Assessment**
      1.  Order 48 units/kg
      2.  Modify order duration to 48 hours or greater
2. **Scenario 2: Intrathecal Chemotherapy Ordering**
   1. **Vignette:** You are scheduled to perform a lumbar puncture with intrathecal chemotherapy in the outpatient procedure center for a patient with B-cell ALL who is undergoing maintenance chemotherapy. Following the lumbar puncture, you will see the patient in the clinic in the afternoon. You have reviewed the patient’s chart. You have verified that signed informed consent is present, and that the patient’s laboratory studies are appropriate to proceed with the lumbar puncture.
   2. **Task:** Perform all necessary tasks within the electronic health record to facilitate the procedure and its documentation.
   3. **Safety risk explanation:**
      1. **Release chemotherapy within the lumbar puncture encounter:** The new EHR requires that providers “release” intrathecal chemotherapy orders so that the administration may be charted within the medication administration record by the scrub nurse in the procedure room. If the provider does not “release” the chemotherapy, it will not appear for the nurse to document as administered. Furthermore, the provider must have the correct encounter selected when “releasing” the medication. If the medication is “released” within the outpatient encounter scheduled for later in the day, for example, the medication will appear for the scrub nurse as “read only” and cannot be documented as administered from within the procedure.
   4. **Assessment:**
      1.  Release chemotherapy within lumbar puncture encounter
3. **Scenario 3: Ewing Sarcoma Chemotherapy Ordering**
   1. **Vignette:** The patient has Ewing sarcoma. He is being admitted to start chemotherapy with vincristine, doxorubicin and cyclophosphamide today. He meets all parameters to start chemotherapy and all pre-treatment evaluations have been completed. Following completion of chemotherapy, his mother will administer pegfilgrastim at home. Another provider has signed the chemotherapy. The orders require a second (verification) signature by another provider, and you have been designated to provide the second signature.
   2. **Task:** Open the chemotherapy orders and perform the necessary tasks to complete the chemotherapy ordering process. A paper copy of the chemotherapy roadmap is presented.
   3. **Safety risk explanation:**
      1. **Modify mesna dose to 360 mg (240 mg/m^2^):** Any orders that include multiple medications in the same bag/syringe (e.g., cyclophosphamide with mesna) require manual dose calculation and entry by providers—they cannot be automatically calculated and entered by the system. We identified manual dose calculation and entry as a high risk task. Our division policy is that all chemotherapy orders must be signed by two providers (or one provider and a pharmacist). In this scenario, the provider who initially ordered the chemotherapy made an error in mesna dose calculation. For a patient with a body surface area of 1.5 m^2^, a dose of 240 mg was ordered instead of 240 mg/m^2^, or 360 mg. The purpose of this scenario was for providers to recognize an incorrect dose ordered in a high-risk scenario during verification of the dose. Recognition within the EHR was made particularly challenging because the doses of cyclophosphamide and mesna were included together on the same line in the order verification screen, and the mesna dose was easy to overlook.
      2. **Release pegfilgrastim:** The EHR required that outpatient take-home prescriptions are both signed and “released” to the pharmacy. Orders that are signed but not “released” are not transmitted to the pharmacy. Providers were not accustomed to this added step in the ordering workflow in the new EHR. In this scenario, the provider who initially signed the chemotherapy failed to “release” the pegfilgrastim after signing the order. The purpose of this scenario was for the provider reviewing the chemotherapy to recognize that the order had not been “released” and to “release” it.
      3. **Notify pharmacy:** Signed orders for chemotherapy do not trigger any notification to the pharmacy to indicate that they are available to be advance verified. Unawareness of orders by the pharmacy leads to unnecessary delays in chemotherapy preparation and administration. We therefore had to develop a workaround to notify the pharmacy of completed chemotherapy orders. The established workaround involves manually sending the pharmacy a message through the EHR’s “inbasket” feature or calling the pharmacy by phone. Providers were unaccustomed to this additional step, and the purpose of this scenario was to reinforce this important step.
   4. **Assessment:**
      1.  Modify mesna dose from 240 mg to 360 mg (240 mg/m^2^)
      2.  Release pegfilgrastim
      3.  Notify pharmacy
4. **Scenario 4: High Dose Methotrexate**
   1. **Vignette:** The patient has high-risk B-cell ALL. He is undergoing chemotherapy which includes high-dose methotrexate. Currently, it is 56 hours following the start of high-dose methotrexate infusion. Urine pH values have been appropriate and he remains on IV fluids at 125 mL/m^2^/hr. His laboratory studies (i.e., methotrexate level and creatinine values) and the high-dose methotrexate level management algorithm are presented in a table.
   2. **Task:** Perform any tasks necessary to ensure good clinical care relating to his chemotherapy.
   3. **Safety risk explanation:**
      1. **Identify leucovorin has not been released:** The new EHR requires inpatient nurses to “release” chemotherapy-related orders so that they may be processed by the pharmacy. If an order is not “released,” it is not processed by the pharmacy and dispensed. By design, orders are “released” on the day in which they are administered. On high-dose methotrexate regimens, leucovorin orders appear on the day they are scheduled to start (in this case, at 42 hours following the start of methotrexate infusion). The nurse caring for the patient at the time leucovorin is due must remember to “release” the order so that it is processed and sent from the pharmacy for administration to the patient. In this simulated scenario, the step was missed and the nurse did not “release” the leucovorin order. The purpose of this scenario was to reinforce that providers must be vigilant about ensuring that leucovorin orders are “released” since this step can easily be overlooked. At the time of the simulations, we were enacting a systems-based change where the nurse will “release” the methotrexate order and a leucovorin order (with a built-in administration delay) at the same time on “Day 1.” We postulated that this would reduce the incidence of unreleased orders. However, implementing this change across all protocol plans involving high-dose methotrexate would take months to complete, so providers were presented with this vignette to encourage vigilance until the new workflow could be built and implemented.
      2. **Identify leucovorin must be re-ordered to continue past hour 54:** The initial build for all high dose methotrexate treatments included leucovorin starting at a prespecified time and administered for a prespecified number of doses, after which the order would be discontinued. If the patient did not meet expected clearance parameters, the provider would need to manually adjust the orders to ensure that leucovorin continued until the patient’s methotrexate level fell to an appropriate level. In our previous EHR configuration, leucovorin would continue every six hours indefinitely until discontinued by the provider. This new workflow posed a risk that providers expected that the leucovorin would continue when, in fact, the order had automatically been discontinued and provider action was required to continue it. In this scenario, the patient required ongoing leucovorin based on methotrexate levels, but the order was set to automatically discontinue. The provider needed to modify the orders to continue leucovorin until methotrexate clearance was achieved. The purpose of this scenario was to remind providers that leucovorin orders automatically discontinue after a specified number of doses. At the time of the simulations, we were enacting a systems-based change where leucovorin orders default setting would be to continue every six hours indefinitely until discontinued by the provider. It was felt that this default (which corresponded to our previous workflow) was safer than the current configuration. However, implementing this change across all protocol plans involving high-dose methotrexate would take months to complete, so providers were presented with this vignette to encourage vigilance until the new workflow could be built.
   4. **Assessment:**
      1.  Identify leucovorin has not been released
      2.  Identify leucovorin must be re-ordered to continue past hour 54
5. **Scenario 5: 6-mercaptopurine Ordering**
   1. **Vignette:** The patient has high-risk B-cell ALL. He is scheduled to be admitted to the hospital tonight to start chemotherapy, including oral 6-mercaptopurine. He meets all laboratory and clinical parameters to start therapy. Paper copies of the chemotherapy roadmap and 6-mercaptopurine dosing table (according to body surface area) are presented.
   2. **Task:** Order chemotherapy for Day 1 of Interim Maintenance
   3. **Safety risk explanation:**
      1. **Place two separate inpatient orders for 6-mercaptopurine utilizing “User Specified” frequency:** In this scenario, the patient required different doses of 6-mercaptopurine to be administered on different days of the week to achieve the target weekly dose. To facilitate this in the inpatient setting, a new ordering workflow was developed whereby users complete two orders—one for each dosage level—and specify on which days patients will be treated at each dosage level (e.g., 50 mg on Sunday through Wednesday and 25 mg on Thursday through Saturday). Because this new workflow required providers to enter an uncommonly-used dosage frequency in the EHR (i.e. “User Specified”) in order to select specific days of the week for the medication to be administered, this scenario allows for providers to practice this new workflow using the uncommon frequency for correct dosage ordering.
      2. **Write 6-mercaptopurine outpatient prescription without conflicting sig:** Similarly, ordering of different dosages on different days of the week presents challenges for the outpatient take-home prescription. Many providers’ standard practice was append the sig with additional instructions. For example, the provider can add to the sig “Take 50 mg on Sunday through Wednesday and 25 mg on Thursday through Saturday. Give last dose on [date].” The default sig within the EHR included a blank dosage that was specified to be taken orally at bedtime for 56 days. This default order included a hard stop which prevented providers from prescribing without specifying a dose in the dose field. This created a risk where providers would enter a dosage equal to the tablet size provided by the manufacturer (i.e., 50 mg) and then append the default sig. When transmitted to the pharmacy, this resulted in a conflicting sig (e.g., “Take 50 mg by mouth at bedtime for 56 doses. Take 50 mg on Sunday through Wednesday and 25 mg on Thursday through Saturday. Give last dose on [date].” The purpose of this scenario was for providers to practice either writing the entire prescription using the free-text feature or to change the dose in the default sig to indicate the range of possible doses and to select a frequency of “as directed” to prevent the transmission of conflicting sigs (e.g., “Take 25-50 mg as directed. Take 50 mg on Sunday through Wednesday and 25 mg on Thursday through Saturday. Give last dose on [date].”).
   4. **Assessment:**
      1.  Place two separate inpatient orders for 6-mercaptopurine utilizing “User Specified” frequency
      2.  Write 6-mercaptopurine outpatient prescription without conflicting sig
